# Rehabilitation of Executive function in Paediatric Traumatic brain injury (REPeaT): protocol for a randomized controlled trial for treating working memory and decision-making

**DOI:** 10.1186/s12887-018-1338-x

**Published:** 2018-11-20

**Authors:** Nikita Sood, Celia Godfrey, Vicki Anderson, Cathy Catroppa

**Affiliations:** 1 Level 4 West, Brain and Mind, Clinical Sciences, Murdoch Children’s Research Institute, 50 Flemington Road, Parkville, VIC 3052 Australia; 2 The Royal Children’s Hospital, Melbourne, Australia; 3 Department of Paediatrics, University of Melbourne, Melbourne, Australia; 4 Department of Psychology, The Royal Children’s Hospital, Melbourne, Australia; 5 Psychological Sciences, University of Melbourne, Melbourne, Australia

**Keywords:** Traumatic brain injury, Paediatric, Working memory, Decision-making, Executive function, Cogmed, Computerized cognitive training, Intervention, Randomized controlled trial (RCT), Functional outcomes

## Abstract

**Background:**

Working memory allows us to hold information in an active state for short periods of time, and is essential in facilitating goal directed cognitive functioning. Difficulties in working memory and decision-making are common post childhood Traumatic Brain Injury (TBI). Despite this, there is a paucity of research pertaining to implementation and effectiveness of interventions to reduce these common difficulties which impact significantly on one’s ability to function independently. One such intervention, Cogmed Working Memory Training Program, has shown success in improving working memory in other childhood clinical populations, but has received little evaluation in the TBI area. This study aims to evaluate whether Cogmed improves working memory and decision-making post childhood TBI and whether these benefits generalize to functional areas.

**Methods:**

The study is a randomized controlled trial (RCT) of the Cogmed (RM version) intervention for children post-TBI. Children aged 7–15 years are initially screened for working memory impairments. Eligible participants are then randomized into either the treatment group (Cogmed) or the active-control group (Lexia Reading). Each group trains online for 50 min each day, 5 days per week, for 5 consecutive weeks. The online training is supported by online clinician meetings each week. Outcome neuropsychological and functional assessments are carried out immediately at the completion of the intervention and at 6 months follow-up.

**Discussion:**

This study follows gold standard methodology in intervention research; uses a novel measure of decision-making; measures the effects of intervention on functional outcomes immediately and longer-term post intervention; uses online clinician support in order to allow more families easy access to the program; and promotes the use of technology to improve health services. If efficacious in improving working memory, decision-making, and functional outcomes, our team will then take a key role in implementing Cogmed into clinical care.

**Trial registration:**

Australian New Zealand Clinical Trials Registry ACTRN12617000085370. Trial Registration Date: 16/01/2017. Protocol Version/Date: HREC 35181G/18.08.2017. Study Status: Ongoing.

## Background

Paediatric Traumatic Brain Injury (TBI) is one of the leading causes of death and disability in children and adolescents worldwide. TBI is defined as “an alteration in brain function, or other evidence of brain pathology, caused by an external force to the head” [1]. Substantial literature on paediatric TBI has identified acute and long-term impairments in higher order cognitive processes often identified as impairments in executive function (EF) [2–4].

Definitions of EF indicate that it encompasses the highest level of human functioning such as working memory (WM), decision-making (DM), aspects of attention, cognitive control, and inhibition [5, 6]. Neural substrates linked to WM and DM include the frontal lobes, prefrontal subregions, posterior cortex, and subcortical structures [7–9] These brain regions mature from childhood into early adulthood with different trajectories across sex and are specifically vulnerable following TBI sustained in childhood [10–12]. Findings from brain imaging studies also suggest that the integrity of these brain regions and the neural circulatory connecting them is crucial for WM [13, 14] and DM [6, 15–17]. With regards to sex, previous research shows difference in performance on WM and DM tasks in girls and boys post childhood TBI [12, 18].

WM is a limited multi-component system that facilitates maintenance and manipulation of information in an active state for short period of times. It is involved in goal-directed cognitive functioning and in the acquisition of academic skills including mathematical computation, reading, and writing [19–24]. Impairments in WM post TBI are common, often with implications in a multitude of cognitive processes and adaptability in daily life [25, 26].

DM is defined as the fundamental skill for identifying, processing, and selecting one course of action from multiple alternatives [27]. Thus, DM is a stepwise process as proposed by the process tracing models of DM [28]. Myriad disciplines have studied DM from different theoretical assumptions ranging from normative theories of subjective expected utility [29], to dual process theories of information processing [30, 31], and to somatic marker hypothesis [32], with less research on process tracing methods of information acquisition in DM [33]. One such method pertains to the use of information boards [34–36]. More recently, an emerging field of decision neuroscience has led to an integrated study of DM by taking into account the neural substrates, cognitive processes, and the association between one’s decisions and social outcomes [4, 37–39]. Findings from these studies suggest WM allows multiple units of information to be assimilated and compared in DM [4, 40]. Additionally, DM has also been associated with functional outcomes such as return to school. In TBI, recent studies have also indicated deficits in DM [41–47]. While there are studies that contradict DM’s association with WM in children with or without brain injuries, findings in support of this association demonstrate a pleading case for more research in this area [48–54].

It is clear that EF domains such as WM and DM provide a target for intervention, given they are frequently impaired post childhood TBI, and are associated with everyday function and social functioning. Previous studies estimate that one-third of children post TBI benefit from intervention approaches [55]. Recently, several computerized interventions or training programs have been successfully trialled for executive function rehabilitation in other clinical populations. Despite this, there is a paucity of research in the intervention area, with even less research pertaining to the effectiveness of interventions implemented following childhood TBI [56, 57]. Methodological limitations of existing research include reliance on case studies instead of RCT design; no preregistration of trials; lack of an active-control group, no blinding, small sample size, and limited use of clinically meaningful outcome measures [58].

The Cogmed Working Memory Training program (Cogmed), is a popular computerized cognitive intervention that is based on the multi-component theory of memory [59] and the theory of neuroplasticity [26]. Cogmed was first trialled in an RCT of 53 children with ADHD [60]. This trial aimed to investigate the impact of Cogmed in improving WM in this clinical population. Results immediately post-intervention and at 3 months follow-up suggested significant benefits in working memory, ADHD symptoms, and several other executive tasks thereby implying the benefits from the intervention were also transferrable. Since then Cogmed has been trialled in typical adults [61], pre-term children [62], children with ADHD, and school-aged children with low WM [63, 64]. However, evidence for benefits from Cogmed in children with TBI is scarce, where to date only seven published studies have investigated the efficiency of Cogmed post brain injury. Of these, six studies involved adults with acquired brain injuries [65–70]. Additionally, limitations of these studies included small sample size, lack of an active-control group, and shorter length of follow-up. A single recent study involving children post-TBI [57] investigated the impact of Cogmed on various components of WM. Findings from this study provided preliminary evidence for the efficacy of Cogmed in improving WM, attention, and academic skills. However, findings are limited by a small sample size, lack of consideration of injury-related factors, and short length of follow-up post intervention. While research investigating the impact of Cogmed in brain injuries supports improvements in similar tasks as those in Cogmed training, other results are inconclusive. [71]. Hence, further research investigating the efficacy of Cogmed in improving performance in similar tasks as well as generalizability to other functional outcomes is required.

In summary, ours is the first study to use a novel task of DM to investigate the relationship between WM and DM, and to study the impact of Cogmed on WM, DM, and functional outcomes in paediatric TBI.

## Study aims

The overall aim of the proposed study is to evaluate the effectiveness of Cogmed post childhood TBI. RCT methodology with the inclusion of an active-control group and a larger sample size will be implemented to address previous limitations in this area. In addition to the primary executive function outcomes post intervention, this trial will investigate the generalized impact (far transfer) of Cogmed in improving everyday function including social functioning. The follow-up assessments will be completed at two time points, namely, immediately post-intervention, and at 6-months follow-up. This RCT will be a novel study to identify the relationship between WM, DM, functional outcomes and the impact of Cogmed in all these areas in children post TBI.

**Fig. 1:**
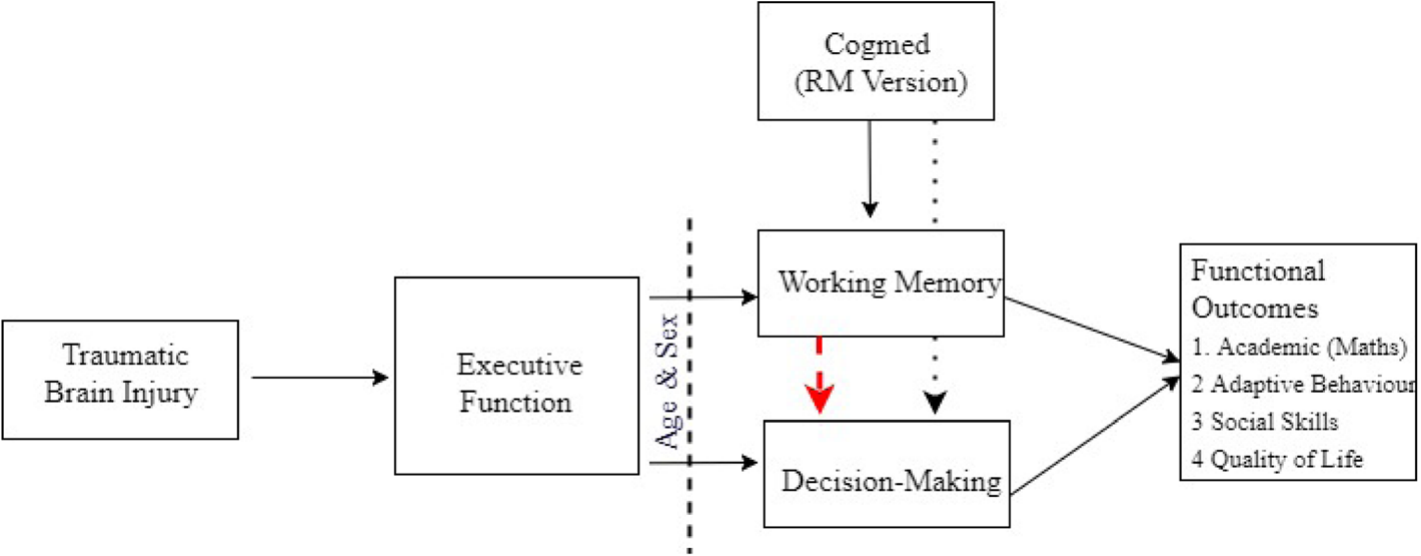
The proposed model of this study

It is hypothesized that: (a) immediately post-intervention and at 6 months post-intervention compared to the active-control group the treatment group will report improvements in WM and DM; (b) improvements in WM will be associated with an increase in DM, academic achievement (mathematics), social skills, behavioural, and quality of life outcomes; and (c) age and sex will have an impact on WM and DM of children post- TBI (see Fig. 1).

## Methods/design

### Approval and registration

This study has been approved by the Human Research Ethics Committee of the Royal Children’s Hospital, Melbourne (HREC 35181), and prospectively registered with the Australian New Zealand Clinical Trials Registry (ACTRN12617000085370).

### Design

**Fig. 2:**
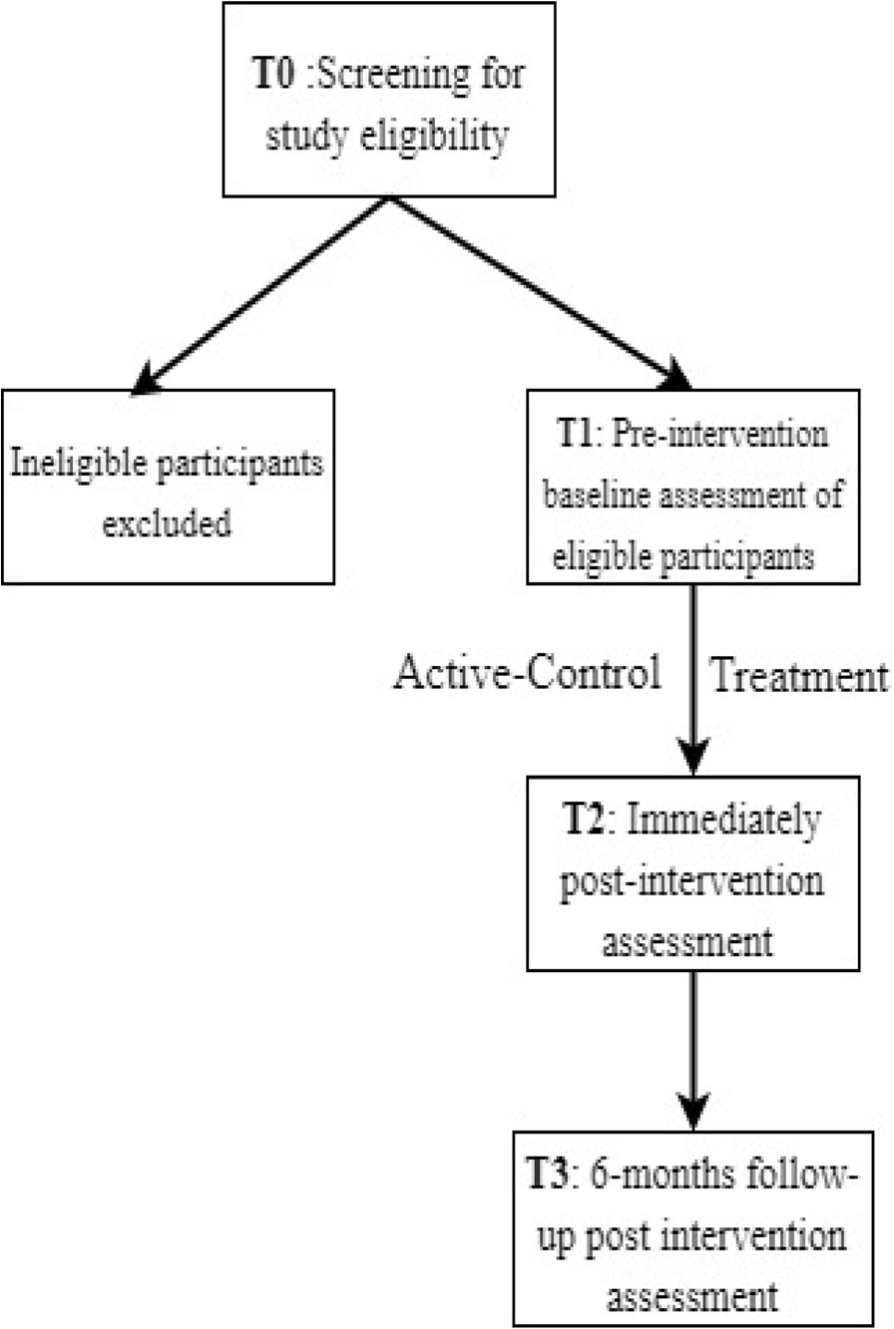
Study design

This study follows the gold standard methodology of intervention research. Conducted and reported according to CONSORT guidelines, it is a double-blinded, active-controlled, randomized trial. Data from neuropsychological assessments of participants, and parent and teacher questionnaires will be collected at four different time-points (see Fig. 2): T0 Study eligibility is determined by screening for working memory impairments in the child at least post 6 months after TBI; T1 Following inclusion of an eligible participant, pre-intervention baseline assessment conducted and data collected; T2 Immediately post-intervention assessment; T3 6-month follow-up post intervention assessment. At all four time points, data will be collected from both the treatment group and the active-control group.

### Setting

This study will be conducted at the Murdoch Children’s Research Institute (MCRI) and The Royal Children’s Hospital (RCH), Australia. Participants will be recruited from The RCH Victorian Paediatric Rehabilitation Service (VPRS) state-wide registry, audit of presentations to The RCH, or from previous research projects conducted at The RCH. The study is expected to run for a 24 month period, with participant identification and recruitment, randomisation, and intervention implementation carried out over 18 months, followed by the 6 month follow-up post-intervention for all the study participants.

### Participants

Children who have had a TBI will be contacted to participate in the study via their parent/guardian. To be eligible to participate in the RCT, children will be required to meet the following inclusion criteria: (1) sustained a TBI where there was head trauma associated with a) altered consciousness, as defined by Glasgow Coma Score between 3 and 15 and/or post traumatic amnesia, or b) intra-cranial traumatic abnormalities on brain scan; (2) evidence of reduced working memory or executive dysfunction at screening (see Table 1); (3) attend primary or secondary school and be between the ages of 7–15 at the time of the intervention; and (4) a minimum of 6 months post-TBI.

**Table 1:** Summarises the study measures and data collection time-points

Outcome	Measure	Respondent	Criteria for inclusion	Time point			
				T0	T1	T2	T3
Screening outcome							
Intellectual functioning	Wechsler Abbreviated Scale of Intelligence-Second Edition (WASI-II) [76]	Child	FSIQ> 70	X			
Working memory	AWMA-Screener [75]	Child	Score on any one of the sub-test < 1 SD below the mean	X			
Executive function	Behavioral Rating Inventory of Executive Function (BRIEF) [77]	Parent	Score on any one of the sub-test < 1 SD below the mean	X			
Primary outcome							
Working memory	AWMA-S [75]	Child			X	X	X
Decision-making	The Decision-Making Task [34]	Child			X	X	X
Secondary outcome							
Working memory	Wechsler Intelligence Scale for Children-Fifth Edition (WISC-V): Digit Span [78]	Child			X	X	X
Decision-making	The Jumping to Conclusions: the Beads Task [79]	Child			X	X	X
Academic achievement (Mathematics)	The Wide Range Achievement Test: Fourth Edition (WRAT-IV): The Math Computation [80]	Child			X	X	X
Adaptive behaviour	Behavioral Rating Inventory of Executive Function (BRIEF): Global Executive Composite [77]	Parent (BRIEF-P) Teacher (BRIEF-T)			X	X	X
	Child Behaviour Checklist (CBCL) & Teacher Report Form (TRF) [81]	Parent (CBCL) Teacher (TRF)			X	X	X
Social skills	Social Skills Improvement System Rating Scales (SSIS) [82]	Parent(SSIS-P) Teacher(SSIS-T)			X	X	X
Quality of life	Paediatric Quality of Life (PedsQL) [83]	Parent			X	X	X

T0: Screening; T1: Pre-intervention baseline assessment; T2: Immediately post-intervention assessment; T3: 6 months follow-up post intervention assessment

Ineligibility for participation in the study will be determined by the following exclusion criteria: (1) non-fluency in English; (2) IQ below 70 at screening (3) other previously documented neurological or learning difficulties diagnosis; (4) severe sensory or physical impairment that affects their capacity to attend mainstream school and complete the training program; (5) families and/or primary caregivers who are unable to support/assist their child through to the completion of the intervention program. This will be determined through discussions with primary caregivers/parents during the recruitment phase.

### Recruitment and allocation

Recruitment will begin with the identification of children who meet the inclusion criteria for a diagnosis of TBI and their current age. Parents/guardians of children will be approached through the tracing letter. After 2 weeks, the research team member will follow-up with a phone-call to provide them with more information about the study. At this time, if the parent/guardian declines their consent for participating in the study, they will be excluded.

When a parent/guardian indicates interest in their child’s participation a letter of invitation to the study will be sent, along with the detailed study information statement and consent form. After the informed written consent is obtained, the first appointment for child’s screening assessment will be arranged at the family’s convenient time. This appointment (T0) will include cognitive screening for working memory impairments and IQ, and if eligible, (T1) the pre-intervention neuropsychological baseline assessment. Eligible participants then will be randomly allocated either to the treatment group or the active-control group.

Parent/guardians will also be provided with the teacher questionnaires along with a reply-paid envelope to forward to their child’s teacher for completion. Teacher consent to participate will be implied in the completion of questionnaires.

### Randomization

Following the first study appointment, eligible participants will be randomized in a ratio of 1:1 to the treatment group or the active-control group. The randomization list will be generated using online software (sealed envelope™) by an independent statistician. Block randomization will be used to generate treatment allocations. At the time of randomization, participants will be allocated the next available sequential study number in an opaque sealed envelope. Allocation of participants to the relevant study group is managed by the chief investigator and will remain blind to the participant, their family and all other members of the trial team. The participants will be also blinded as to which program they have been assigned. However once the program is commenced treatment allocation may become clear to participating families.

### Intervention delivery

Participants in the treatment group will be administered Cogmed ™ (RM version) program. Developed for children aged 7 years and above, it is a commercially available [72] online adaptive working memory training program. For the purpose of this study, participants will train on the standard protocol consisting of a total 25 training blocks to be completed over a span of 5 weeks. Participants will be required to complete one training block in 50 min every day for 5 days a week for 5 weeks. Each training block includes a series of interactive and adaptive exercises that not only targets but also dynamically adapt to the visuo-spatial and verbal WM ability of the participants at the time of the training. During the training, if the participant makes consecutive errors on four trials, the program will automatically enforce a mandatory break for 15 s. At the completion of each training block, the participant gets rewarded with a reward game, RoboRacing.

Cogmed training will be completed online by the participants from their homes. It will be supervised by a trained Cogmed Coach who is the study co-ordinator. Once the participants are randomized to the treatment group, the Cogmed Coach will conduct a Start-up session with the participants and their Training Aides. In this session, the Cogmed Coach will (a) provide an information sheet to set up the program on the home computer or iPad, (b) explain the training structure, (c) plan the training times (d) discuss participants’ motivation and expectations, (e) discuss reward system, (f) schedule online weekly meetings, and (g) fill out the Cogmed Questionnaire. Online meetings (through Skype Business/Google Hangouts/GoTo Meetings) will be held once per week for 5 weeks of training to provide support and answer any queries.

The training performance and compliance will be monitored through a secure server by the Coach.

The program requires internet access and if necessary families will be provided with an internet-connected iPad for the duration of the program. The coach can monitor the training and compliance by logging into a secure server. Program compliance can be assessed as the number of sessions completed by each child, and time spent per session will be recorded.

### Implementation of active control*:* Lexia Reading Core5

Participants in the active-control group will be administered commercially available Lexia Reading Core5 [73, 74]. An adaptive computerized reading program, Lexia battery caters for all age groups of children but has no memory training component. Consisting of a total 18 level and 89 activities, it delivers structured, in-depth, and individualized training in six areas of reading – Phonological Awareness, Structural Analysis, Vocabulary, Phonics, Fluency, and Comprehension. Each participant will first complete 20 min of auto-placement on their initial log-in to Lexia Reading Core5. Auto-placement is a tool that assesses participant’s current level of functioning. Based on this performance, participants will be assigned an appropriate program level for their training. Once participants successfully complete a prescribed set of units, they will progress to higher levels of the program. In addition to the online training, Lexia also provides supplementing individualized paper-based practice materials for participants struggling on a particular activity. On successful completion of a level, the participant is rewarded with a certificate. Participants will train on Lexia Reading Core5 for 50 min each day for 5 days a week for 5 weeks at their homes.

The Lexia program provides an active- control for the experience of sitting in front of a computer and engaging with fun learning tasks randomized to the participants in the active-control group. As with Cogmed, the coach will provide assistance with the set-up and weekly online meetings.

### Technology

Since this RCT uses online training programs and online clinician support, internet enabled iPads will be provided to the participants with no access to a computer. Technical issues faced by the participant will be addressed by the project co-ordinator in the online weekly meetings.

### Facilitator training and program fidelity

The Chief Investigator and the study co-ordinator were trained in administration and use of Cogmed and Lexia. Program fidelity will be monitored through the Coach’s weekly online meetings with families.

### Study measures

The participants will be screened for eligibility in a neuropsychological assessment (approximately 1–2 h). If the child is found eligible for the intervention study, questionnaires covering background and demographic information as well as behavioural function, will be completed by parents/guardians (approx. 1.5–2 h) and the teacher. The study measures are all widely-used, standardised tests and questionnaires appropriate for the target age group, and have established reliability and validity. Details pertaining to the participant’s injury (e.g., date of injury, age at injury, diagnosis, Glasgow Coma Score, neurological signs) are extracted from the hospital database. The parents/guardian provides the demographic information.

WM and DM of the participants are the two primary outcomes for this study. Automated Working Memory Assessment (AWMA) [75] is a validated computerized measure of WM skills, is used for assessing WM. AWMA- Short (AWMA-S) comprises of four sub-tests, namely, Listening Recall (verbal WM), Spatial Recall (visuo-spatial WM), Digit Recall (verbal short-term memory), and Dot Matrix (visuo-spatial short-term memory). In this battery, WM tasks involve both storage and processing of information whereas short-term memory tasks involve storage with minimal processing.

DM is measured using an experimental digital task that has been conceptualised on the basis of a previous research study [34]. Initially, the child is presented with everyday scenarios for example, purchasing a book. They are presented with different aspects related to that scenario, for example, cost of the book. Then they are asked to identify from choices presented on an information board what they feel is most important in making that decision. In order to reach the final decision in a situation, information acquisition is a key process in DM. Therefore, by understanding how children process information in this task, this study purports to further the knowledge of underlying cognitive processes involved in DM.

Secondary outcomes include functional measures of academic achievement, social, behavioural, and quality of life outcomes. Parent and teacher report measures will be administered to assess social and behavioural status, and quality of life outcomes.

### Participant flow and estimated sample size

**Fig. 3:**
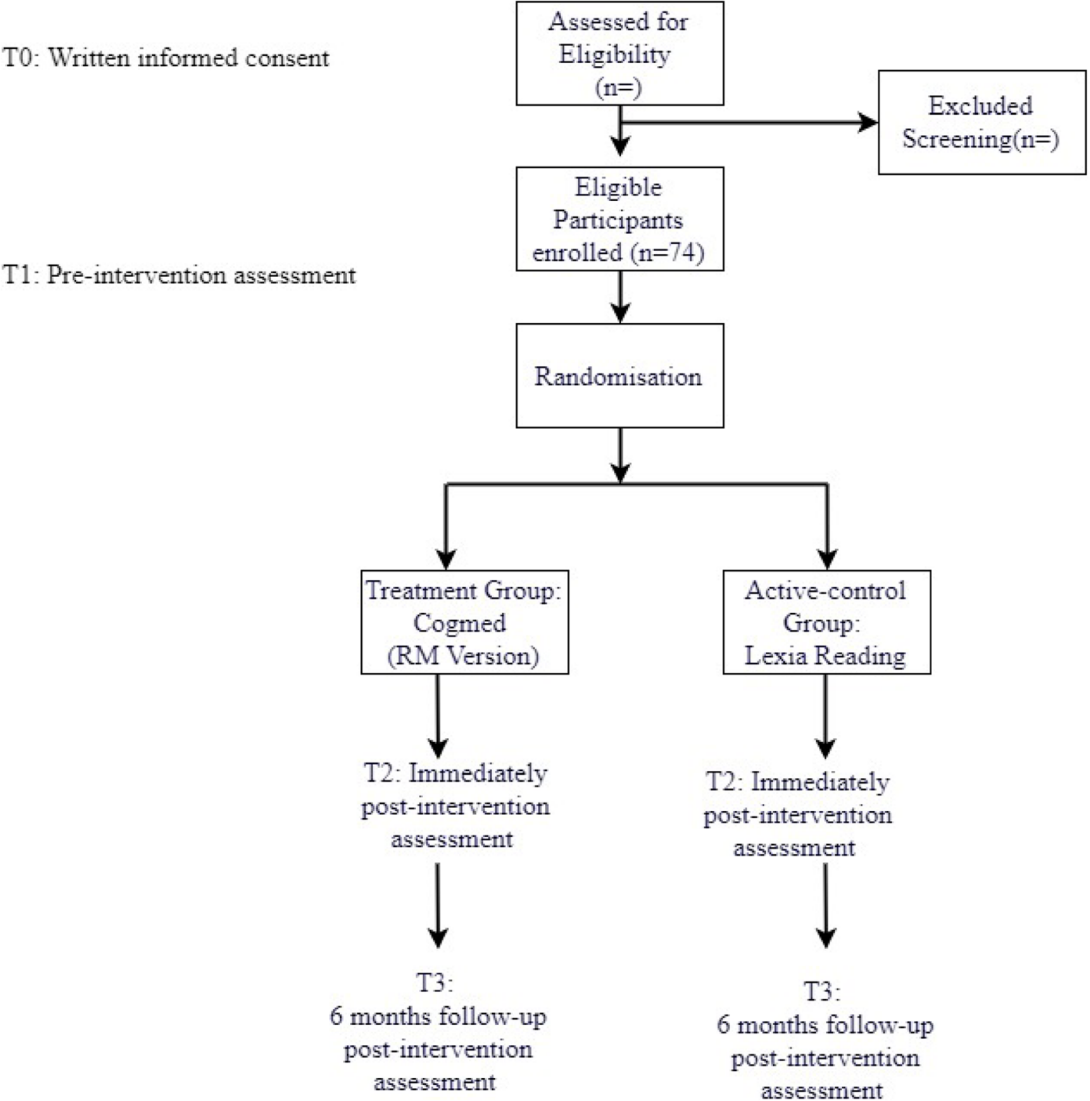
Represents the estimated participant flow and sample size

This study will recruit 74 eligible participants and randomize 37 participants each in the treatment group and the active-control group. This estimated sample size is in accordance with our power calculation so that a clinically significant difference can be found between the treatment group and the active-control group. Sample size calculations were based on the ability to detect a statistically significant difference of 0.8 SD between the treatment group and an active control group based on Automated Working Memory Assessment, with a significance level of 0.05 and power of 0.80 (Fig. 3).

### Data storage

Data will be collected in both pen-paper and digital format and securely stored at MCRI. Only this study’s chief investigators will have access to the collected data, both throughout and after the conclusion of the study.

### Data analysis

This RCT will follow the intention-to-treat paradigm for all the study participants. In order to compare pre-, immediately post-, and 6 months follow-up post intervention performance on the outcome measures all questionnaire and assessment data will be analysed using the SPSS General Linear Model procedure. Primary analysis will be done using simple t-statistics or chi-square statistics to evaluate differences between groups. Secondary analysis will involve explore the effect of moderators on working memory and decision-making. .

### Trial management

This RCT will be supervised by chief investigators CC and CG who will meet fortnightly to discuss the study status. They will also manage unexpected occurrences relating to the protocol or any unforseen adverse events. The RCH Ethics Committee and the Australian New Zealand Clinical Trials Registry will be notified if necessary. No harm, potential or actual, is anticipated as a consequence of participation in this study, but in case of severe distress to any study participant, appropriate clinical referrals will be made.

### Strengths and limitations

This study has notable strengths. The trial will follow the gold-standard methodology in intervention research, i.e., double-blind, randomized-controlled, pre-registered with clinical trials registry, inclusion of active-control group, large sample size, and long duration of follow-up. There is limited research on DM, the relationship between WM and DM, and the efficacy of Cogmed in a paediatric TBI population. This study will address these gaps in the literature. DM deficits are typically assessed using tasks that are limited in their ability to replicate everyday functioning. A novel measure of DM has been developed in order to measure naturalistic decision-making processes in children. To our knowledge, this is the first trial investigating the impact of Cogmed on WM, DM, and functional outcomes in TBI population. The use of online training programs and online clinician support will ensure fewer burdens on families, higher participation, and adherence to training.

Predicted challenges of this study include recruitment of such a large sample size with respect to a screening component, ensuring participant adherence to training regardless of randomisation, and participant follow-up**.** Another limitation pertains to the blinding of participating families. While the research personnel responsible for screening participants and administering outcome assessments are completely blinded, it is not possible to completely blind the parents to the training programs.

## Discussion

Impairments in WM and DM commonly occur following childhood TBI. Currently there is minimal literature and a poor evidence base regarding intervention in this area. It is essential that effective treatment options are made available. The expected outcome of this proposed study is that the Cogmed program will be shown to be effective in improving WM and DM, and will also lead to generalised improvements in functional skills in children post-TBI. Our team will play a key role in translating Cogmed into standard clinical care, schools, and community settings. However, if findings are not significant, it will still be significant in knowing that Cogmed may not be an effective intervention for this population, suggesting it may need to be adapted for implementation in this clinical group.

## Abbreviations

ADHD: Attention deficit hyperactivity disorder
AWMA-S: Automated working memory assessment-short
BRIEF-P: Behaviour rating inventory of executive function-parent
BRIEF-T: Behaviour rating inventory of executive function-teacher
CBCL: Child Behaviour Checklist
DM: Decision-making
EF: Executive function
FSIQ: Full scale intelligence quotient
HREC: Human resources ethics committee
MCRI: Murdoch Children’s Research Institute
PedsQL: Paediatric Quality of Life
RCH: Royal Children’s Hospital
RCT: Randomized controlled trial
SD: Standard deviation
SSIS-P: Social skills improvement system rating scales-parent
SSIS-T: Social skills improvement system rating scales- teacher
T0-T2: Data collection time points 0 to 2
TRF: Teacher Report Form
VPRS: Victorian paediatric rehabilitation services
WASI-II: Wechsler Abbreviated Scale of Intelligence- Second Edition
WISC-V: Wechsler Intelligence Scale For Children- Fifth Edition
WM: Working memory
WRAT-IV: Wide Range Achievement Test: Fourth Edition
